# Exploring the Protective Effect and Mechanism of *Buddlejae Flos* on Sodium Selenite-Induced Cataract in Rats by Network Pharmacology, Molecular Docking, and Experimental Validation

**DOI:** 10.1155/2022/7776403

**Published:** 2022-05-14

**Authors:** Xian-Yin Liu, Xue-Lin Wang, Mo-Chang Qiu, Ying-Jun Ye, Fang Wang, Yan-Qi Xu, Jun-Jie Gong, Zi-Jin Xu

**Affiliations:** ^1^Department of Pharmacy, Jiangxi Medical College, Shangrao 334000, China; ^2^Affiliated Ophthalmic Hospital, Jiangxi Medical College, Shangrao 334000, China; ^3^Pharmacy Department, Yiwu Central Hospital, Yiwu 322000, China

## Abstract

**Objective:**

*Buddlejae Flos* has a long history of utilization by humans to treat ophthalmic diseases. Although *in vitro* study revealed that it can be used for treating cataract, the bioactive components and the mechanism of efficacy remained unclear. This study aims to discover the bioactive components and mode of efficacy of *Buddlejae Flos* in cataract treatment.

**Methods:**

Several databases were screened for bioactive components and corresponding targets, as well as cataract-related targets. Using the String database, common targets were determined and utilized to construct protein-protein interactions (PPI). The drug-component-target-disease network map was drawn using Cytoscape software. *R* language was utilized to execute Kyoto Encyclopedia of Genes and Genomes (KEGG) and Gene Ontology (GO) pathway enrichment analysis. Molecular docking was done through Schrödinger Maestro software utilization. Luteolin's (LUT) effect on cataract induced by sodium selenite in rat pups was evaluated.

**Results:**

Six bioactive components with 38 common targets were identified as being associated with cataract. TP53, AKT1, EGFR, CASP3, TNF, ESR1, INS, IL6, HIF1A, and VEGFA were identified as core targets in PPI analysis, and the binding energy of LUT with AKT was the lowest. LUT has been demonstrated to significantly lower MDA levels, raise glutathione (GSH) levels, and boost the activity of antioxidant enzymes like GST, SOD, GPx, and CAT. After LUT treatment, TNF-a, IL-2, and IL-6 levels were significantly lowered. Bcl-2 mRNA expression levels and p-PI3K and p-AKT protein expression were significantly elevated. In contrast, caspase-3 and Bax mRNA expression levels were significantly decreased.

**Conclusion:**

This study demonstrates that LUT is a possible bioactive component that may be utilized for cataract treatment. Its mode of action includes oxidative stress suppression, reducing inflammation, and inhibiting apoptosis via regulating the PI3K/AKT single pathway.

## 1. Introduction

According to previously published American Academy of Ophthalmology (AAO) clinical guideline, cataract is an ophthalmic disease characterized by visual impairment due to degradation of lens optical properties, which is highly age-related [1]. A meta-analysis of the influence of different regions and ages on the prevalence of cataract indicated that the global total prevalence of cataract was 17.20%, with 36.55% in southeast Asia and 9.08% in America, and 54.38% of patients were over 60 years old [2]. More than 10 million people worldwide are blind due to cataract, accounting for 40% of all blind people, and up to 90% in developed countries. Another 35 million people have moderate to severe vision impairment due to cataract [3–5]. It can be observed that cataract affects more than one billion people globally, more than half of whom are elderly, and it has become a social problem in China and even the whole world. Due to the unique advantages of a novel intraocular lens in material, design, and optical properties, surgery has become the primary treatment option for cataract [6, 7]. However, it should not be ignored that cataract surgery has a series of complications such as incision infection, posterior capsular rupture, posterior capsular rupture, capsular contraction syndrome, intraocular lens dislocation, and dry eyes [8, 9]. In addition, the high cost of cataract surgery limits its application in many less developed countries. Therefore, developing drugs for cataract prevention and treatment is greatly significant.

Previous research has revealed that cataract has been categorized according to their etiology as age-related, congenital, and juvenile, or secondary to disease, trauma, drug, ultraviolet (UV), and associated factors such as smoking and drinking. Several studies have also disclosed that cataract may be associated with high blood pressure, obesity, autoimmune diseases, chronic kidney disease, and diabetes [10, 11]. In the study of senile cataract, cataract caused by oxidative stress, systemic diseases, trace element deficiency, and glucose metabolism disorder has attracted extensive attention [12]. Therefore, the existing cataract treatment drugs mainly include antioxidant drugs (glutathione, L-cystine, lutein, zeaxanthin, vitamin E/C, carotenoids, etc.), anti-aldose reductase drugs (benzyl lysine and diosgenin), and crystallin-dissolution drugs (lanosterol and rosmarinic acid) [13]. With further studies on cataract pathogenesis, the oxidative stress role in forming and developing cataract has been further clarified [14–16].

It is worth mentioning that about 44 medicinal plants/natural items have been utilized for treating cataracts in various traditional and folk medicine systems, including ayurveda, traditional Chinese medicine (TCM), and Korean traditional medicine [17]. *Buddlejae Flos* has a long history of utilization in TCM for treating ophthalmic diseases because of its effect on clearing heat and purging fire, nourishing the liver, and brightening eyesight (TCM terminology). The previous study has demonstrated that flavonoids or their glycosides in 70% methanol extracts of *Buddlejae Flos* exhibited significant aldose reductase inhibitory activity *in vitro*, suggesting that its possible components may be a potential cataract treatment drug [18]. However, the bioactive components and potential mechanisms of *Buddlejae Flos* in cataract treatment have not been elucidated, limiting its clinical application.

In this study, network pharmacology was first utilized for screening bioactive components and corresponding targets of *Buddlejae Flos*. GO enrichment, KEGG enrichment, and PPI were utilized to further analyze the common targets of *Buddlejae Flos* and cataracts. The corresponding targets and bioactive components were then screened using molecular docking, and the results were finally validated by the sodium selenite-induced cataract model in rats.

## 2. Methods

### 2.1. Screening of Bioactive Components of *Buddlejae Flos*

The Traditional Chinese Medicine Systems Pharmacology (TCMSP, https://tcmspw.com/tcmspsearch.php) database was employed for screening the bioactive components of *Buddlejae Flos* [19]. In accordance to ADME, drug similarity (DL) > 0.18 and oral bioavailability (OB) 30% were established as threshold for screening (absorption, distribution, metabolism, and excretion) parameters [20, 21]. To ensure reliable and comprehensive data, data collected from published literature were used to complement findings from TCMSP [22–24].

### 2.2. Bioactive Component-Related Target Collection

The Swiss Target Prediction database was utilized to collect corresponding targets of bioactive components in *Buddlejae Flos* with “*Homo sapiens*” species setting and TCMSP database (http://www.swisstargetprediction.ch/). To merge search results and delete duplicate records, the UniProt database was utilized (https://www.uniprot.org/).

### 2.3. Cataract-Related Target Collection

GeneCards database (http://www.genecards.org/), Online Mendelian Inheritance in Man database (OMIM, http://www.omim.org/), and DisGeNET database (https://www.disgenet.org/) were utilized to search for potential targets linked to cataract using the search term “cataract” (UMLS CUI : C0029531). Additionally, the known targets of drugs for treating cataract recorded in the DrugBank database (https://www.drugbank.ca/), including PTGS1 and PTGS2, were collected for further analysis.

### 2.4. Construction of the Network between *Buddlejae Flos* and Cataract-Related Targets

The Venn diagram developed by the online tool Venny 2.1 was utilized to visualize the common targets between *Buddlejae Flos* bioactive components and cataract-related targets (https://bioinfogp.cnb.csic.es/tools/venny/). Cytoscape software (version 3.7.2) was utilized to visualize the drug-component-target-disease network. The String database (https://string-db.org/cgi/input.pl) was utilized for establishing PPI network for common targets with a minimum requisite interaction score of >0.4 setting. PPI network's parameters, including betweenness centrality (BC), node degree (degree), and closeness centrality (CC), were computed and presented in a histogram and 3D scatter diagram.

### 2.5. GO and KEGG Pathway Enrichment Analyses

The “clusterProfile” *R* package was utilized to accomplish GO and KEGG enrichment analyses while *P*-value < 0.05 or *Q*_Value_ < 0.05 was considered to be significantly enriched. *R* packages “Enrichlot” and “ggplot2” were then utilized to visualize enrichment findings, which were showcased in a bar chart and a bubble chart. Finally, the core targets in the graphics of critical signaling pathways were highlighted in red using “Pathview” *R* package. *R* software 3.6.2 (x64) was utilized to accomplish all preceding stages [25].

### 2.6. Molecular Docking of Bioactive Compounds of *Buddlejae Flos* with Core Common Targets

Molecular docking of core bioactive compounds (luteolin, apigenin, and acacetin) and core common targets (AKT1, TP53, CASP3, EGFR, and TNF) were performed using Schrödinger Maestro software suite (version 9.1, Schrödinger, L.L.C.) [26, 27]. Briefly, the PubChem database was utilized to download the molecular structures of core bioactive components (https://pubchem.ncbi.nlm.nih.gov/). After minimizing energy and optimizing by Schrodinger software, the standard pdpqt files were saved as a ligand. The PDB database was utilized to download the protein structures of core common targets (http://www.rcsb.org/). After removing water and other unrelated molecules, the protein structures were processed using Schrodinger software with a protein preparation wizard module via hydrogenation, calculation of charge, and combination of nonpolar hydrogen, and finally saved as a receptor. Subsequently, the grid box coordinates were set, and the active site was determined in accordance with each protein's natural ligand. The box size was set as 40 × 40 × 40 grid points, with a distance of 0.1 nm between the tiny grid points. Finally, using default software parameters, the core bioactive compounds were docked with the common core targets by flexible docking. The complex compound with protein was visualized using Pymol software (version 2.1, DeLano Scientific LLC.).

### 2.7. Experimental Validation

#### 2.7.1. Materials

From Nanjing Dasf Biotechnology Co. Ltd, LUT was purchased (purity ≥98%) (Nanjing, China). Sodium selenite (purity ≥99%) was purchased from Sigma-Aldrich (Shanghai) Trading Co., Ltd (Shanghai, China). The Nanjing Jiancheng Bioengineering Institute provided the following kits: superoxide dismutase (SOD), malondialdehyde (MDA), reduced glutathione (GSH), glutathione S-transferase (GST), glutathione peroxidase (Gpx), catalase (CAT), tumor necrosis factor-*α* (TNF-*α*), interleukin-2 (IL-2), and interleukin-6 (IL-6) (Nanjing, China). TIANGEN Biotech provided the Fast Quant RT Kit (Beijing, China). YiShan Biotech provided a RNA-Quick Purification Kit (Shanghai, China). Zen bioscience (Chengdu, China) provided the GAPDH antibody and HRP-Goat anti-Mouse, while Abcam provided anti-PI3K, anti-AKT, anti-p-PI3K, and anti-p-AKT (Cambridge, UK). All other chemicals were of analytical grade.

#### 2.7.2. Animals

Grade SPF Sprague-Dawley rat pups (7-day-old, both male and female, body weight 18 ± 2 g, Shanghai Slac Laboratory Animal CO., Ltd. SCXK 2012-0002) were maintained in a clean grade facility at the Jiangxi Medical College's Experimental Animal Center (temperature 22 ± 2°C, relative humidity 45%∼65%). All animals were kept on 12-hour light/12-hour darkness cycles and had unrestricted access to tap water and standard rat food (Trophic Animal Feed High-tech Co. Ltd., Nantong, China). Jiangxi Medical College's Animal Protection Research Ethics Committee authorized the study (2020090401).

#### 2.7.3. Animal Treatment

After adaptive feeding for three days, 10-day-old SD rats were allocated to five groups randomly (in every group *n* = 10). Rats in the model group and three treatment groups with different doses of LUT were injected subcutaneously with sodium selenite solution (2.46 mg·kg^−1^, injection volume no more than 0.5 mL) in the cervical region at 10, 12, and 14 days of age while rats administered a subcutaneous injection of normal saline simultaneously. From 10 days of age, for three weeks, the rats in control and model groups received normal saline by oral gavage. LUT received by oral gavage to rats in low-dose (L-LUT), medium-dose (M-LUT), and high-dose (H-LUT) groups at dosages of 50, 100, and 200 mg·kg^−1^, respectively.

#### 2.7.4. Lens Opacity Score

After 3-week treatment, the lens opacity was observed and photographed for records. The score of lens opacity was evaluated by five independent researchers according to the following principles: each rat was recorded for lens opacity on a scale of 0 to 4, with half-steps of 0.5, where score 0 represents no apparent opacity and score 4 represents opacity of more than 75% of the lens's cross-sectional area (complete cataract) [28].

#### 2.7.5. Sample Collection

Half an hour after the last oral gavage, the rats were anesthetized with isoflurane, and the aqueous humor from both eyes was collected and pooled using a microinjector. Subsequently, all rats were sacrificed by prompt dislocation of the neck vertebra under deep anesthesia with isoflurane. Lens and retinas from both eyes were isolated, washed three times in ice-cold phosphate buffer saline (PBS). The aqueous humor and lens samples were kept at −80°C until they were analyzed, while the retina samples were kept in 10% neutralized formalin for subsequent paraffin embedding and histological examination.

#### 2.7.6. Measurement of Oxidative Stress Biomarkers in Lens

Lens samples were immersed in PBS and homogenized for 60 s at 60 Hz with a tissue homogenizer (Scientz-48, Ningbo, China). The supernatant was collected by homogenate centrifugation for 10 min at 3500 r/min at 4°C. The BCA protein concentration determination kit was utilized for measuring total protein concentration. MDA and GSH levels, as well as the activity of GST, SOD, CAT, and GPx, were quantified using commercial kits in accordance with manufacturer's instructions.

#### 2.7.7. Measurement of Inflammatory Biomarkers in Aqueous Humor

TNF-*α*, IL-2, and IL-6 levels in aqueous humor were quantified through enzyme-linked immunosorbent assay (ELISA) kit usage as directed by manufacturer's instructions.

#### 2.7.8. Histological Analysis

The histopathology of the retina was evaluated using hematoxylin and eosin staining (H&E). The retina was embedded in paraffin, cut into 4 *μ*m segments, and stained with H&E for 48 h of fixation in 10% neutralized formalin. Under an optical microscope, the specimens' histological diagnostic and microscopic characteristics were observed and photographed (Olympus IX81, Japan, magnification, × 400).

#### 2.7.9. RT-PCR Analysis

RNA-Quick Purification Kit was utilized for the total mRNA of lens extraction. Subsequently, from 2 *μ*g total RNA, cDNA was synthesized and utilized for RT-PCR. The following RT-PCR was performed: Step 1 : 95°C for 10 min, Step 2 : 95°C for 10 s, and Step 3 : 72°C for 30 s for 39 cycles. As an internal control, glyceraldehyde-3-phosphate dehydrogenase (GAPDH) was utilized. The LUT effect on relative mRNA gene expression was computed utilizing 2^−ΔΔcq^ method. The primer sequences utilized in this study are summarized in Table 1.

#### 2.7.10. Western Blot Analysis

Total protein was isolated from the lens and utilized for evaluating p-AKT, PI3K, AKT, and p-PI3K protein expression levels. The BCA protein assay kit was utilized to determine the total protein concentration. Thereafter, SDS-PAGE with tris-glycine SDS was utilized as the running buffer and the proteins were separated before being transferred to a PVDF membrane at 80 mA for 1 h utilizing tris-glycine SDS transfer buffer mixed with 20% methanol. In turn, the membrane was blocked with 5% skimmed milk and incubated with antibodies overnight (PI3K: 1 : 5000; AKT: 1 : 1000; anti-p-PI3K: 1 : 1000, anti-p-AKT: 1 : 500; GAPDH: 1 : 5000) on a 4°C cradle. Subsequently, following rinsing of the membrane with TBST, secondary antibodies were incubated with the membrane for 1 h at 37°C (1 : 5000). Following that, the membranes were stained with a western bright dye to allow faster and reversible identification of protein bands. Image *J* software was utilized to quantify protein bands.

### 2.8. Statistical Analysis

The mean standard deviation (SD) of all data was computed and analyzed utilizing SPSS software (version 19.0). One-way analysis of variance (ANOVA) was performed for comparing different groups, and *P* < 0.05 was deemed statistically significant.

## 3. Results

### 3.1. Bioactive Components of *Buddlejae Flos*

TCMSP database was utilized for 46 bioactive components examinations. However, only four of them met the screening criteria, including butyrospermyl acetate, linarin, luteolin, and acacetin. Additionally, another three bioactive components were identified according to the published articles listed above, including hesperetin, apigenin, and acteoside. Butyrospermyl acetate was excluded from further analysis due to unclear chemical abstracts service (CAS) number and the absence of any disease and target association information in the TCMSP database. Detailed information on the six bioactive components involved in the subsequent analysis is listed in Table 2.

### 3.2. Network between *Buddlejae Flos* and Cataract-Recorded Targets

**Figure 1:**
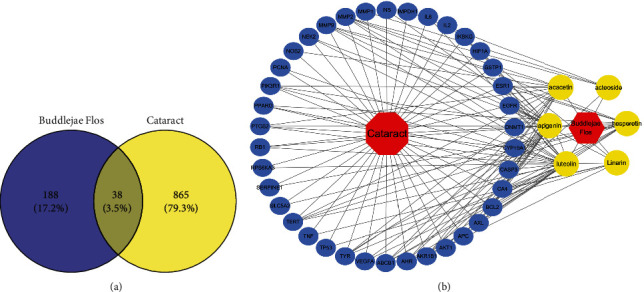
(a) Bioactive component-related targets and cataract-related targets in the Venn diagram. (b) Drug-component-target-disease network.

TCMSP and Swiss Target Prediction databases were utilized for collecting 174 and 423 corresponding targets of bioactive components, respectively. After standardization and removing duplicates using the UniProt database, 226 targets were recognized as bioactive components related targets for subsequent analysis. Cataract-related targets were obtained from multiple databases, such as 49 targets from OMIM database, 579 targets from DisGeNET database, 6719 targets from GeneCards database, and 2 targets from DrugBank database. After cleaning the data by de-duplication, 903 targets were recognized as cataract-related targets. A Venn diagram (Figure 1(a)) shows the overlap of bioactive component-related targets and cataract-related targets. A total of 38 common targets were identified, and their details are illustrated in Table 3. The drug-component-target-disease network was constructed as illustrated in Figure 1(b). The findings indicated that LUT interfered with 25 of 38 common targets, followed by apigenin (24), acacetin (16), hesperetin (13), linarin (7), and acteoside (4).

### 3.3. PPI Network of Common Targets

**Figure 2:**
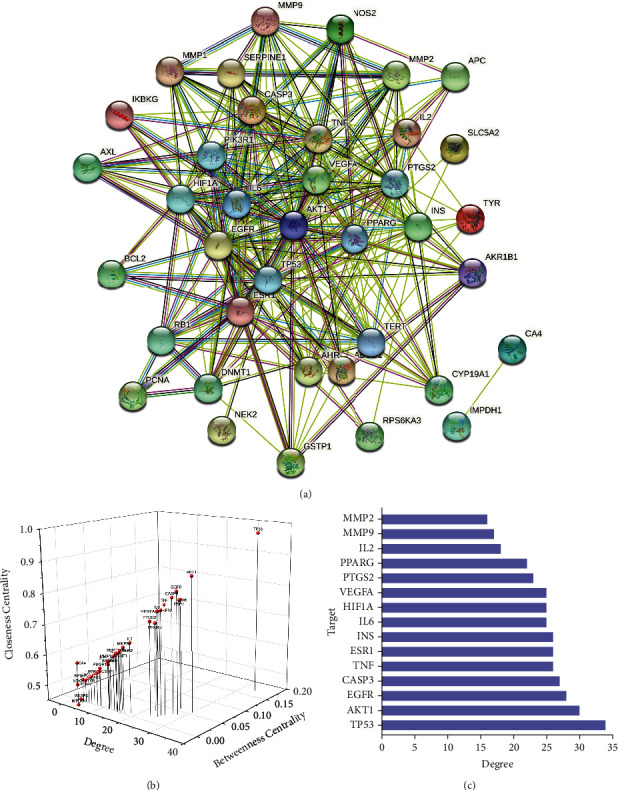
(a) PPT network of common targets. (b) 3D scatter diagram of the key parameters of all nodes. (c) Bar chart of a degree value.

**Figure 3:**
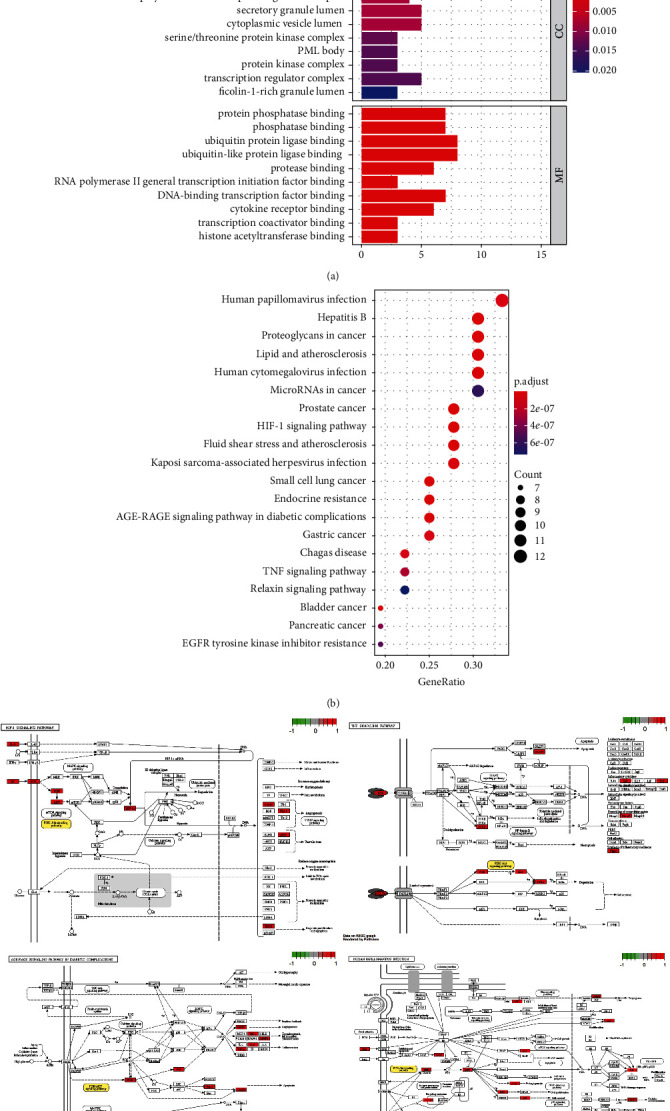
(a) Bar chart of GO enrichment analysis. (b) Bubble chart of KEGG analysis. (c) Representative signaling pathways maps.

PPI network, with 38 nodes and 283 edges, was established based on the common targets of bioactive component-related targets and cataract-related targets (Figure 2(a)). The BC, degree, and CC of all nodes in PPI network were depicted in the form of a 3D scatter diagram. As displayed in Figures 2(b) and 3(c), TP53, AKT1, EGFR, CASP3, TNF, ESR1, INS, IL6, HIF1A, VEGFA, PTGS2, PPARG, IL2, MMP9, and MMP2 were the top 15 core targets.

### 3.4. GO and KEGG Enrichment Analysis

The 38 common targets of GO enrichment analysis are illustrated in Figure 3(a). The findings illustrate that the top predictors in biological processes (BP) include response to oxidative stress, response to light stimulus, response to UV, and cellular response to chemical stress. In terms of cellular components, transferase complex, phosphorus-containing groups vesicle lumen, transcription regulator complex, secretory granule lumen, and cytoplasmic vesicle lumen were significantly enriched. Additionally, a molecular function was significantly enriched for ubiquitin-protein ligase binding, ubiquitin-like protein ligase binding, protein phosphatase binding, phosphatase binding and, DNA-binding transcription ligase binding. KEGG enrichment analysis revealed that infection-related signaling pathways, inflammation-related signaling pathways, apoptosis signaling pathways, and oxidative stress-related signaling pathways were significantly enriched in diabetes-related complications, like human papillomavirus infection, hepatitis, Kaposi sarcoma-associated herpesvirus infection, TNF-signaling pathway, HIF-1 signaling pathway, and AGE-RAGE signaling pathway (Figure 3(b)). Representative signaling pathways maps are illustrated in Figure 3(c), with the positions of core targets in the signaling pathways highlighted in red. More comprehensive analysis reveals that PI3K/AKT signaling pathway (highlighted in yellow) was implicated in all enriched signaling pathways and was situated at a crucial position in the pathway, compatible with PPI results.

### 3.5. Molecular Docking Analysis

**Figure 4:**
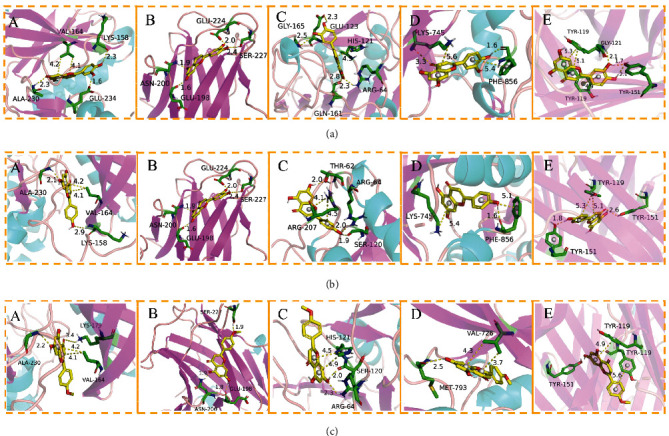
(a) Binding mode of luteolin with AKT1, TP53, CASP3, EGFR, and TNF. (b) Binding mode of apigenin with AKT1, TP53, CASP3, EGFR, and TNF. (c) Binding mode of acacetin with AKT1, TP53, CASP3, EGFR, and TNF. The protein's backbone was rendered in a tube and coloured in bright blue. Compounds are rendered by yellow. A, B, C, D, and E represent AKT1, TP53, CASP3, EGFR, and TNF, respectively. The yellow dash indicates the hydrogen bond distance or *π*-stacking distance.

**Figure 5:**
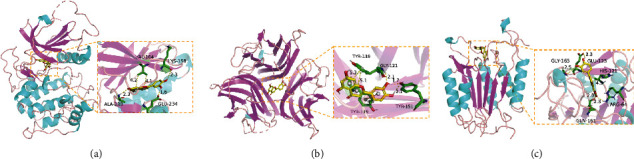
(a) Binding mode of luteolin with AKT1. (b) Binding mode of luteolin with TNF. (c) Binding mode of luteolin with CASP3. Using the yellow dashed lines, we indicate that the right columns were bigger than the left columns.

The docking results of core bioactive compounds (luteolin, apigenin, and acacetin) and core common protein targets (AKT1, TP53, CASP3, EGFR, and TNF) are shown in Table 4. The binding energies are lower than -6.0 kcal/mol, indicating that the binding conformations of components with proteins are stable. The binding modes of the protein with bioactive compounds are clearly displayed in Figures 4 and 5. Notably, the active groups of LUT can form hydrogen bonds with the active groups of LYS-158, ALA-230, and GLU-234 of AKT1, with hydrogen bond distances were 2.3 Å, 2.3 Å, and 1.6 Å, respectively. LUT has a stronger binding force with AKT1 because hydrogen bond distance is shorter than that of traditional hydrogen bond (usually around 3.5 Å); these hydrogen bond interactive plays a crucial role in the stability of binding conformations. In addition, the *σ*-*π* conjugated interaction between the benzene ring of LUT and Val-164 (a hydrophobic amino acid of AKT1) were observed, indicating strong hydrophobic interaction between LUT and the active pocket of proteins, contributing to conformation stability. Overall, all this evidence shows that LUT has the unique advantages in the effective interaction with protein target and is likely to be a potential bioactive component of *Buddlejae Flos* in cataract treatment by interfering with AKT-related signaling pathways.

### 3.6. Experimental Validation

#### 3.6.1. Lens Opacity Score

**Figure 6:**
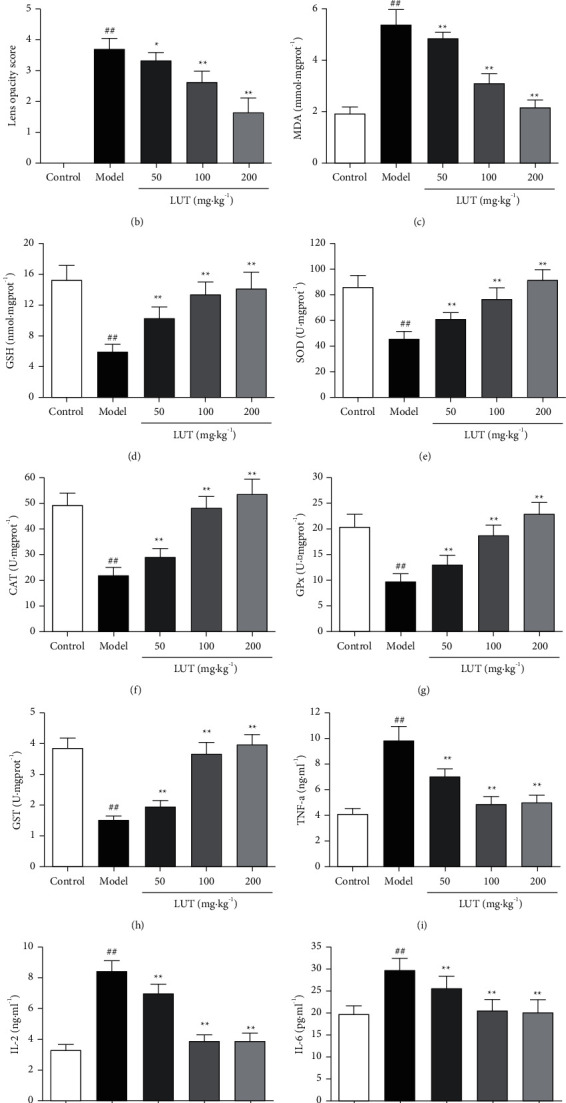
Effect of LUT on lens opacity, biomarkers of oxidative stress, and biomarkers of inflammation (*n* = 10). (a) Photographs of lens opacity. (b) Lens opacity score. (c) MDA. (d) GSH. (e) SOD. (f) CAT. (g) GPx. (h) GST. (i) TNF-*α*. (j) IL-2. (k) IL-6. ^##^*P* < 0.01 vs. control group; ^*∗∗*^*P* < 0.01 vs. model group.

As displayed in Figures 6(a) and 6(b), the images obtained show a significantly difference of the opacity of the lens among the groups. In comparison to the control group, the model group's lens opacity score was significantly elevated, revealing that the cataract model was established successfully. In M-LUT and H-LUT groups, rats' lens opacity score was significantly reduced compared to the model group, demonstrating that LUT might ameliorate the lens opacity caused by cataract.

#### 3.6.2. Effects of LUT on Oxidative Stress Biomarkers

As illustrated in Figures 6(c)–6(h), the level of MDA was significantly increased, while GSH level, SOD, CAT, GPx, and GST activities were significantly reduced in comparison to the control group, suggesting that oxidative stress level was significantly elevated following sodium selenite treatment. After 3-week LUT treatment, all biomarkers of oxidative stress were improved with statistical significance in all dose groups. The results revealed that LUT could improve sodium selenite-induced cataract by lowering oxidative stress level.

#### 3.6.3. Effects of LUT on Biomarkers of Inflammation

In comparison to the control group, TNF-*α*, IL-2, and IL-6 concentrations in aqueous humor of the model group were significantly elevated, indicating that the inflammation level was significantly elevated following sodium selenite treatment. After 3-week LUT treatment, TNF-*α*, IL-2, and IL-6 concentrations in all dose groups were significantly decreased comparing to the model group. The results revealed that three doses of LUT could significantly mitigate the level of inflammation (Figures 6(i)–6(k)).

#### 3.6.4. Histological Analysis of Retina

**Figure 7:**
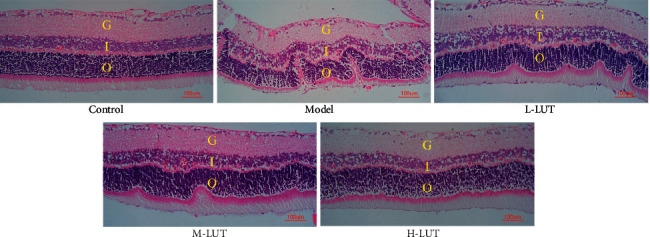
Representative photomicrographs of H&E staining (× 400). G I, O represent the Ganglion cell layer, inner nuclear layer, and outer nuclear layer, respectively.

Histological examination of the retina using H&E staining is presented in Figure 7. A homogenous surface of the retina with a regular arrangement of cells at the ganglion cell layer, inner nuclear layer, and the outer nuclear layer was observed in the control group. Meanwhile, swelling of the retina with a disordered cell arrangement in each layer after sodium selenite induction was observed in the model group. The distribution of ganglion cell inner and outer nuclear layers was improved in LUT treatment groups compared to the model group.

#### 3.6.5. Effects of LUT on the mRNA Expression of Caspase-3, Bax, and Bcl-2

**Figure 8:**
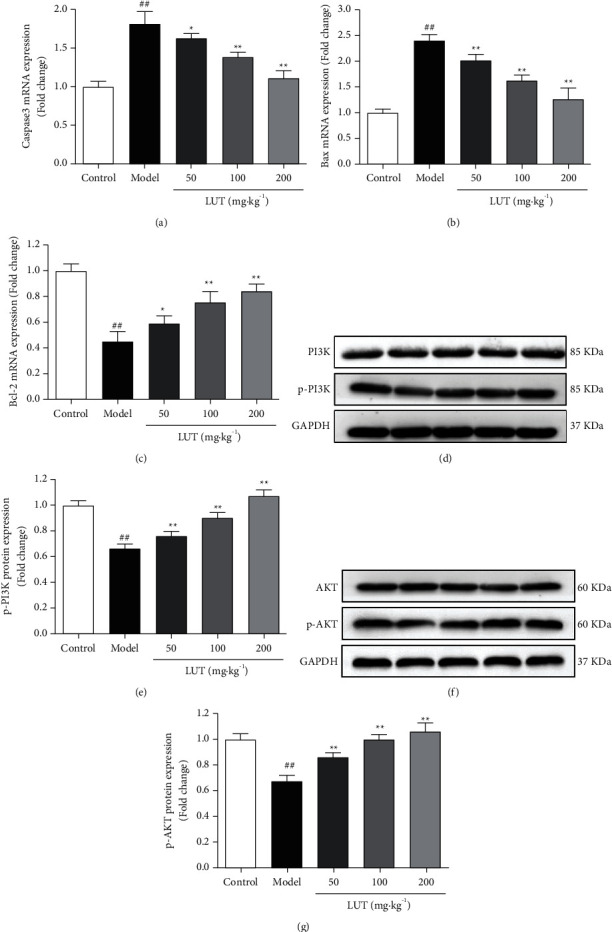
Effect of LUT on the levels of mRNA and protein expression (*n* = 3). (a) Caspase-3. (b) Bax. (c) Bcl-2. (d) PI3K and p-PI3K Western blot analysis. (e) Densitometry examination of p-PI3K. (e) AKT and p-AKT Western blot analysis. (f) Densitometry examination of p-AKT. ^##^*P* < 0.01 vs. control group; ^*∗*^*P* < 0.05 vs. model group; ^*∗∗*^*P* < 0.01 vs. model group.

Caspase-3 and Bax mRNA expression levels were significantly elevated in the model group, whereas Bcl-2 expression levels were significantly lowered in comparison to the control group. As illustrated in Figures 8(a)–8(c), caspase-3 and Bax mRNA expression levels were significantly lowered in all dosage groups, whereas Bcl-2 expression levels were significantly elevated in comparison to the model group.

#### 3.6.6. Effects of LUT on the Protein Expression of PI3K, AKT, p-PI3K, and p-AKT

Western blotting analysis was utilized for studying the protein expression levels of PI3K, AKT, p-PI3K, and p-AKT in the lens. As depicted in Figure 8(d)–8(g), p-PI3K and p-AKT protein expression levels in the model group were significantly lowered in comparison to the control group and were significantly raised in all dose groups in comparison to the model group. In PI3K and AKT expression levels, there was no significant variation among the five groups, indicating that LUT activates the PI3K/AKT signaling pathway by increasing PI3K and AKT phosphorylation.

## 4. Discussion

In this study, the initial step was to discover the bioactive components and targets of *Buddlejae Flos* in cataract treatment using network pharmacology and molecular docking. The most inspiring aspect of the present study is that LUT has been identified as a potential bioactive component in *Buddlejae Flos* and may play a therapeutic role in cataract by intervening PI3K, AKT, caspase-3, Bax, Bcl-2, TNF-*α*, IL-2, and IL-6. This conclusion was ultimately confirmed using a sodium selenite-induced cataract model in rats. The *Buddlejae Flos* mechanism in treating cataract includes oxidative stress suppression, inhibiting apoptosis, and mitigating inflammation by regulating the PI3K/AKT single pathway.

Recently, network pharmacology has been increasingly utilized in the study of TCM to greatly accelerate the discovery of drugs and clarify the mechanism of action [29–31]. However, applying network pharmacology in studying ophthalmic diseases and ophthalmic herbs is very rare. The results of network pharmacology confirm that Qing Guang An Granule regulates p53, HIF-1, PI3K-Akt, and neurotrophin signaling pathways to treat glaucoma, which is similar to our results [32]. VEGFA, AKT1, and IL-6 were recognized as core targets in the network pharmacology prediction study of *Astragalus membranaceus* in treating the diabetic retinopathy, and the PI3K-Akt signaling pathway role has also been highlighted [33]. The association between *Buddlejae Flos* and cataract was investigated for the first time in the current study by the molecular docking, and the network pharmacology was conducted to reveal binding conformations of bioactive components and targets. Flavonoids exhibit unique advantages in molecular docking due to their abundant active functional groups, which are more likely to form stronger hydrogen bonds with the binding pocket amino acid residues of the protein [34, 35]. The stable conformations were formed in previous molecular docking studies between LUT and target proteins (such as EGFR, HAS, and NLRP3) due to the abundance of hydrogen bonds in the structure [36–38]. In the current study, an obvious binding advantage was observed between LUT and five target proteins. It is speculated that LUT has one more hydrophilic hydroxyl group structurally than apigenin and acacetin, which changes the hydrophobic properties of flavonoids to some extent.

Previous studies have revealed that PI3K transmits essential signals that regulate an assortment of physiological processes in virtually every type of tissue studied to date, including inflammation, cancer, immune deficiency, tissue overgrowth, and cellular metabolism [39, 40]. AKT is the most widely studied PI3K signaling effector, and it impacts most of phenotypes linked with PI3K pathway activation [41]. Notably, in various disease models, oxidative stress and apoptosis are linked to the PI3K/AKT signaling pathway [42–44]. In recent years, the pathogenesis of diabetic cataract has been further elucidated, which may be related to changes in intraocular permeability, oxidative stress, and crystalline protein glycosylation [45]. However, the pathogenesis of senile cataract remains unclear, and it is the mainstream theory that aging-related oxidative stress aggravates and then induces the increase of insoluble lens protein leading to cataract [46]. Furthermore, GO and KEGG enrichment results in this study focused on oxidative stress, inflammation, and apoptosis-related pathways. Therefore, it is inferred that cataract development and occurrence are both influenced by the PI3K/AKT signaling pathway, which has also been confirmed in previous studies [47, 48]. The rat model of cataract induced by sodium selenite was further used for validation experiment [49]. Biomarkers of oxidative stress (MDA, GSH, GST, SOD, CAT, and GPx), inflammation (TNF-a, IL-2, and IL-6), and apoptosis (caspase3, Bax, and Bcl-2) were significantly improved after LUT treatment, consistent with the results of a previous study on curcumin for cataract treatment [50]. Additionally, retinal dysfunction secondary to cataract has been observed in similar studies [51]. The result of retina histological demonstrated that LUT hindered cataract development in lenses and improved retinal function. Previous evidence reveals that PI3K/AKT signaling pathway activation contributes to growth, differentiation, and injury repair of lens epithelial cells [52]. In this study, the protein expressions of p-PI3K and p-Akt were significantly increased after LUT treatment, which may be the molecular mechanism of LUT treatment for cataract.

## 5. Conclusions

In conclusion, our study demonstrates that LUT is a potential bioactive component of *Buddlejae Flos* which is capable of treating cataract. Its mode of action includes oxidative stress suppression, alleviating inflammation, and preventing apoptosis via regulating the PI3K/AKT single pathway.

## Figures and Tables

**Table 1:** Primers utilized for mRNA expression analyses.

Gene	Primer sequences	
GAPDH	Forward	5′- TGGCTGTTAGTGTGTCAGGC -3′
	Reverse	5′- CTTCCGGGAGGTTCCATCTG -3′

Caspase3	Forward	5′- CCGATGTCGATGCAGCTAAC -3′
	Reverse	5′- CTTTCCAGTCAGACTCCGGC -3′

Bcl-2	Forward	5′-CACAGAGGGGCTACGAGTG -3′
	Reverse	5′-AGCGACGAGAGAAGTCATCCC -3′

Bax	Forward	5′- TGGGGGTCTGTTTGCTTTAGG -3′
	Reverse	5′- CTACTGCTTCTGATGGACAGGG -3′

**Table 2:** Detailed information on the six bioactive components in *Buddlejae Flos*.

Mol Id	CAS	Molecule name	Molecular formula	MW	OB (%)	DL	Structure
MOL001790	480-36-4	Linarin	C_28_H_32_O_14_	592.60	39.84	0.71	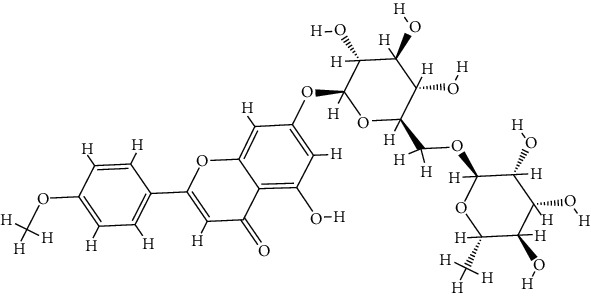

MOL000006	491-70-3	Luteolin	C_15_H_10_O_6_	286.25	36.16	0.25	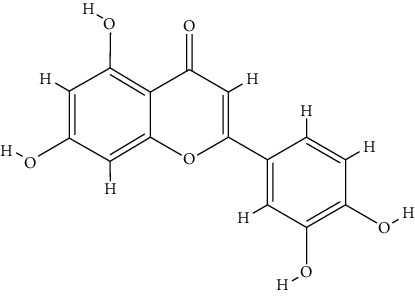

MOL001689	480-44-4	Acacetin	C_16_H_12_O_5_	284.28	34.97	0.24	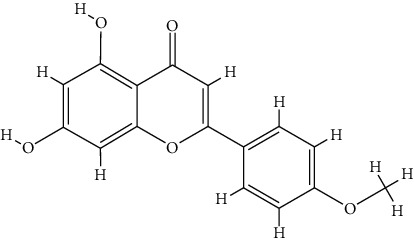

MOL002341	520-33-2	Hesperetin	C_16_H_14_O_6_	302.30	70.31	0.27	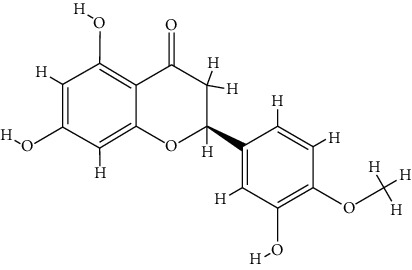

MOL000008	520-36-5	Apigenin	C_15_H_10_O_5_	270.25	23.06	0.21	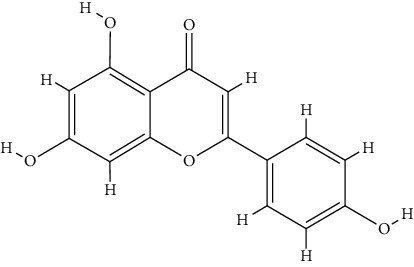

MOL003333	61276-17-3	Acteoside	C_29_H_36_O_15_	624.65	2.94	0.62	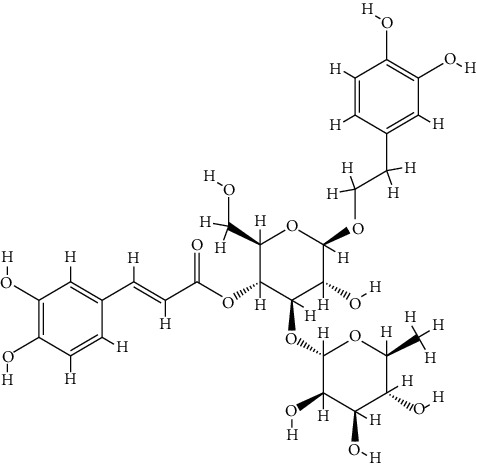

**Table 3:** Detailed information on common targets.

No.	Uniprot ID	Target gene	Target protein	Major molecular function
1	P05231	IL6	Interleukin-6	Cytokine
2	P12004	PCNA	Proliferating cell nuclear antigen	DNA-binding
3	P09211	GSTP1	Glutathione S-transferase P	Transferase
4	P15692	VEGFA	Vascular endothelial growth factor A	Growth factor
5	P06400	RB1	Retinoblastoma-associated protein	Repressor
6	P42574	CASP3	Caspase-3	Protease
7	Q16665	HIF1A	Hypoxia-inducible factor 1-alpha	Activator
8	Q9Y6K9	IKBKG	NF-kappa-B essential modulator	DNA damage
9	P01308	INS	Insulin	Hormone
10	P25054	APC	Adenomatous polyposis coli protein	Wnt signaling pathway
11	P11511	CYP19A1	Aromatase	Oxidoreductase
12	P03372	ESR1	Estrogen receptor	Receptor
13	P35354	PTGS2	Prostaglandin G/H synthase 2 ·	Oxidoreductase
14	P08183	ABCB1	ATP-dependent translocase ABCB1	Translocase
15	P22748	CA4	Carbonic anhydrase 4	Lyase
16	P14780	MMP9	Matrix metalloproteinase-9	Hydrolase
17	P14679	TYR	Tyrosinase	Oxidoreductase
18	P35869	AHR	Aryl hydrocarbon receptor	Receptor
19	O14746	TERT	Telomerase reverse transcriptase	Nucleotidyltransferase
20	P00533	EGFR	Epidermal growth factor receptor	Host cell receptor for virus entry
21	P30530	AXL	Tyrosine-protein kinase receptor UFO	Receptor
22	P27986	PIK3R1	Phosphatidylinositol 3-kinase regulatory subunit alpha	Host-virus interaction
23	P51955	NEK2	Rine/threonine-protein kinase Nek2	Transferase
24	P31749	AKT1	RAC-alpha serine/threonine-protein kinase	Transferase
25	P35228	NOS2	Nitric oxide synthase, inducible	Calmodulin-binding
26	P01375	TNF	Tumor necrosis factor	Cytokine
27	P60568	IL2	Interleukin-2	Growth factor
28	P51812	RPS6KA3	Ribosomal protein S6 kinase alpha-3	Transferase
29	P04637	TP53	Cellular tumor antigen p53	Repressor
30	P05121	SERPINE1	Plasminogen activator inhibitor 1	Protease inhibitor
31	P31639	SLC5A2	Sodium/glucose cotransporter 2	Ion transport
32	P26358	DNMT1	DNA (cytosine-5)-methyltransferase 1	Chromatin regulator
33	P37231	PPARG	Peroxisome proliferator-activated receptor gamma	Receptor
34	P10415	BCL2	Apoptosis regulator Bcl-2	Apoptosis
35	P08253	MMP2	72 kD a type IV collagenase	Hydrolase
36	P15121	AKR1B1	Aldo-keto reductase family 1 member B1	Oxidoreductase
37	P20839	IMPDH1	Inosine-5′-monophosphate dehydrogenase 1	Oxidoreductase
38	P03956	MMP1	Interstitial collagenase	Hydrolase

**Table 4:** Docking results of core bioactive compounds and core common targets.

Targets	Compounds	Binding Energy (kcal/mol)	Combination type
Luteolin	AKT1	−8.58	Hb, Hi, *π*-stacking
Luteolin	TP53	−7.99	Hb, Hi
Luteolin	CASP3	−8.21	Hb, Hi, *π*-stacking
Luteolin	EGFR	−7.98	Hb, Hi, *π*-stacking
Luteolin	TNF	−8.44	Hb, Hi, *π*-stacking
Apigenin	AKT1	−7.86	Hb, Hi, *π*-stacking
Apigenin	TP53	−7.7	Hb, Hi
Apigenin	CASP3	−7.98	Hb, Hi, *π*-stacking
Apigenin	EGFR	−8.01	Hb, Hi, *π*-stacking
Apigenin	TNF	−7.4	Hb, Hi, *π*-stacking
Acacetin	AKT1	−7.35	Hb, Hi, *π*-stacking
Acacetin	TP53	−7.49	Hb, Hi
Acacetin	CASP3	−7.16	Hb, Hi, *π*-stacking
Acacetin	EGFR	−7.98	Hb, Hi, *π*-stacking
Acacetin	TNF	−8.07	Hb, Hi, *π*-stacking

“Hb” represents hydrogen bonds and “Hi” represents hydrophobic interactive.

## Data Availability

All datasets used to support the findings of this study are included within this study.
